# Immune biology of NSCLC revealed by single-cell technologies: implications for the development of biomarkers in patients treated with immunotherapy

**DOI:** 10.1007/s00281-022-00973-1

**Published:** 2022-11-21

**Authors:** J. Wlosik, S. Fattori, P. Rochigneux, A. Goncalves, D. Olive, A. S. Chretien

**Affiliations:** 1 Team Immunity and Cancer, Centre de Recherche en Cancérologie de Marseille (CRCM), CNRS UMR7258, Institut Paoli-Calmettes, Aix-Marseille University UM105, Inserm U1068, 13009 Marseille, France; 2 Immunomonitoring Department, Institut Paoli-Calmettes, 13009 Marseille, France; 3 Department of Medical Oncology, Inserm U1068, Aix-Marseille University UM105, CNRS UMR7258, Institute Paoli-Calmettes, 13009 Marseille, France; 4 Team Cell Polarity, Cell Signaling and Cancer, Centre de Recherche en Cancérologie de Marseille (CRCM), CNRS UMR7258, Institut Paoli-Calmettes, Aix-Marseille University, Inserm U1068UM 105, 13009 Marseille, France

**Keywords:** Single-cell technologies, Predictive biomarkers, NSCLC, Immunotherapy, irAEs

## Abstract

First-line immunotherapy in non-small-cell lung cancer largely improved patients’ survival. PD-L1 testing is required before immune checkpoint inhibitor initiation. However, this biomarker fails to accurately predict patients’ response. On the other hand, immunotherapy exposes patients to immune-related toxicity, the mechanisms of which are still unclear. Hence, there is an unmet need to develop clinically approved predictive biomarkers to better select patients who will benefit the most from immune checkpoint inhibitors and improve risk management. Single-cell technologies provide unprecedented insight into the tumor and its microenvironment, leading to the discovery of immune cells involved in immune checkpoint inhibitor response or toxicity. In this review, we will underscore the potential of the single-cell approach to identify candidate biomarkers improving non-small-cell lung cancer patients’ care.

## Biomarker identification in advanced non-small-cell lung cancer (NSCLC): a crucial need

Lung cancer is the leading cause of cancer death worldwide [1]. In the last decade, the development of immune checkpoint inhibitors (ICI) has significantly improved clinical outcomes in advanced NSCLC [2–5]. Unfortunately, the pattern of ICI response is extremely heterogeneous, from hyper-progressors [6] to durable responders, [7] with the majority of patients not deriving significant benefits from ICI therapy [8]. Furthermore, ICI have a broad spectrum of toxicity and significant cost [9, 10]. Consequently, identifying robust predictive biomarkers is crucial but remains challenging despite numerous efforts.

Tumor programmed death ligand 1 (PD-L1) immunohistochemistry assay is used to determine the proportion of PD-L1 expressing tumor cells. PD-L1 expression was the first ICI biomarker to be published [2] and is currently the only one used in clinical routine. Multiple prospective trials have demonstrated a correlation between the level of tissue PD-L1 expression and clinical efficacy [11], notably KEYNOTE-001 [2], KEYNOTE 024 [12] and PACIFIC trial [13]. The Blueprint PD-L1 IHC Assay Comparison Project evaluated the diagnostic performances of 4 commercially available assays used in clinical trials, since each assay uses a specific clone for their monoclonal primary antibody: 22C3 for pembrolizumab, 28–8 for nivolumab, SP263 for nivolumab and SP142 for atezolizumab. 22C3, 28–8 and SP263 assays had similar performances but SP142 showed a decreased sensitivity [14]. However, the optimal nature of IHC staining (tumor cells, immune cells or both) and the cut-off value of PD-L1 staining positivity is still being debated. Moreover, some studies showed that PD-L1 expression failed to predict clinical outcomes under ICI therapy [15] and some patients with < 1% PD-L1 expression have substantial response rates [16]. For these reasons, PD-L1 expression is considered an imperfect biomarker that needs to be challenged. On the other hand, tumor mutational burden (TMB) quantifies the number of somatic mutations per coding area of a tumor genome using whole exome sequencing (WES) or FoundationOne CDx assay. TMB at the 10 mut/mb cut-point was approved by FDA in 2020 as a biomarker for pembrolizumab benefit across tumor types in the USA [17]. However, TMB faces several limitations in NSCLC such as the arbitrary threshold [18] or the absence of a consistent survival benefit of the TMB high phenotype [19, 20]. Notably, TMB failed to demonstrate its predictive value in NSCLC patients from the Checkmate 227 trial treated with nivolumab plus ipilimumab [21].

To date, PD-L1 expression and TMB are the only biomarkers used to stratify patients in prospective phase 3 clinical trials, whereas other biomarkers are still investigational. For instance, PD-L1 expression on tumor-infiltrating lymphocytes (TILS) was found to be strongly associated with outcome [22]. Despite being associated with objective response rate (ORR) and progression-free survival (PFS) in patients treated with pembrolizumab, lymphocyte infiltration did not substantially add to the predictive value of PD-L1 expression alone for overall survival (OS) [23]. In the blood, baseline Lung Immune Prognostic Index, combining derived neutrophil to lymphocyte ratio and lactate dehydrogenase level was associated with OS in NSCLC patients treated with ICI [24]. Finally, numerous potential predictive biomarkers of ICI efficacy are currently being studied: (i) T-effector and INF-γ-related gene signature [25]; (ii) peripheral blood markers such as proliferating PD-1^+^CD8 T cells [26] or serum interleukin-8 [27]; (iii) circulating tumor cells [28] or miRNAs/exosomes [29]. Most of these biomarkers still need a prospective validation to challenge PD-L1 expression in clinical routine.

## Relevance of single-cell technologies in the identification of predictive biomarkers for immunotherapy in NSCLC

PD-L1 expression remains the only biomarker used to predict ICI response in clinical routine. However, the increasing knowledge on immune checkpoints shed the light on the complexity of their regulation [30], and accumulating evidence highlights the weakness of relying on one single molecule to predict treatment responsiveness. Thus, the ideal biomarker is still to be discovered and might result in a combinative approach [31] with more robustness to predict clinical benefits from immunotherapy. Over the years, the rising era of single-cell technologies [32, 33] unveiled the possibility to dissect a tumor and its microenvironment, including immune cells, with surgical precision by targeting multiple parameters at once. Multiple layers of information, spanning from surface protein expression to transcriptomic changes, can be integrated to have a comprehensive understanding of the mechanisms driving immunotherapy responses and, therefore, offering an opportunity to identify future biomarkers. Hereinafter, we will pinpoint the relevance of single-cell technologies in the identification of predictive biomarkers for immunotherapy in NSCLC (Table 1).

**Table 1:** Summary of the reviewed studies using single-cell technologies for the identification of predictive biomarkers for immunotherapy in NSCLC

Reference	Authors	Single-cell technology	Samples	Number of markers	Number of cells	Type of cells	Key findings
[36]	Datar et al. (2019)	Mass cytometry	*N* = 20 primary resected NSCLCs	35	n.d	TILs	High expression of LAG-3 on T cells is associated with a poorer outcome after PD-1 inhibitors and might confer increased resistance to immunotherapy Low expression of LAG-3 and high tumor PD-L1 expression are associated with an increased progression-free survival after immunotherapy
[40]	Kagamu et al. (2020)	Mass cytometry	*N* = 126 blood samples from NSCLC patients	43	20,000	T cells	Nivolumab responders have increased frequencies of circulating CD62L^low^ CD4^+^ T cells whereas non-responders display more regulatory T cells
[44, 45]	Simoni et al. (2018) Yeong et al. (2021)	Mass cytometry	*N* = 50 patients with lung tumor	31	n.d	CD8^+^ TILs	Tumor-specific CD39^+^CD8^+^ TILs can predict response to immunotherapy
[48]	Gueguen et al. (2021)	scRNA-seq	*N* = 11 primary NSCLCs from untreated patients	-	28,936	CD3^+^ TILs	Circulating and tumor-resident CD8^+^ T cells with memory-like features differentiate into terminal effectors
		sTCR-seq	*N* = 6 primary NSCLCs from untreated patients	-	19,508		
[53]	Banchereau et al. (2021)	Mass cytometry	*N* = 6 freshly resected NSCLCs	38	~ 48,000	CD8^+^ T cells	Tumors enriched with CD103^+^CD8^+^ tissue-resident memory T cells have increased sensitivity to atezolizumab
		scRNA-seq		-	26,951		
		sTCR-seq		-			
[59]	Patil et al. (2022)	Mass cytometry	*N* = 6 freshly resected NSCLCs		~ 48,000	B cells	Increased expression program of plasma cell predicts response to atezolizumab
		scRNA-seq	*N* = 11 lung adenocarcinomas		45,149		
[62]	Leader et al. (2021)	CITE-seq	*N* = 8 NSCLCs patients	15–81	361, 929	CD45^+^ cells	Increased frequencies of PD-1^+^CXCL13^+^ activated T cells, SPP1^+^ monocyte-derived macrophages, IgG^+^ plasma cells and a high TMB are predictive of enhanced response to atezolizumab
		scRNA-seq	*N* = 35 NSCLCs patients	-	n.d		
		sTCR-seq	*N* = 3 NSCLCs patients	-	n.d		
[63]	Lau et al. (2022)	scRNA-seq	*N* = 10 samples of frozen dissociated tumor cells	-	62,723	CD45^+^ cells	Combining TMB and a 25-genes signature related to cytotoxicity can predict response to immunotherapy, regardless of HLA-I disruption
[66]	Li et al. (2022)	scRNA-seq	*N* = 10 Stk11/Lkb1 mutant allografts	-	n.d	CD45^+^ cells	Axl inhibitor in mutant Stk11/Lkb1 mice lacking TCF1^+^PD-1^+^CD8^+^ T cells improves pembrolizumab response
		sTCR-seq		-	n.d		
[71]	Jia et al. (2021)	scRNA-seq	*N* = 1 case of pseudoprogression under immunochemotherapy	-	4,858	TILs	First case report of pseudoprogression under neoadjuvant immunochemotherapy caused by immune infiltration
		Imaging mass cytometry		13	-		
[74]	Larroquette et al. (2022)	Spatial transcriptomics	*N* = 16 NSCLCs	-	n.d	CD163^+^ TILs	High TAM infiltration is associated with resistance to immunotherapy

n.d.: no data

### *Mass cytometry*

Deep immunophenotyping of a large number of cells at a single-cell resolution was made possible by mass cytometry. The samples are stained with antibodies conjugated to heavy-metal isotopes, which prevents spectral overlap observed with flow cytometry. Furthermore, the number of parameters that can be monitored considerably increased, up to 40–60 markers in daily practice [34], allowing for both identification and functional characterization of multiple cell subtypes at the protein level. These technological advances substantially improved our understanding of immune responses and provided the opportunity to uncover pathological alterations affecting the immune system in cancer and resistance mechanisms limiting the success of immunotherapies.

T cells expressing inhibitory immune checkpoints are hyporesponsive upon antigen stimulation and classified as exhausted [35]. Datar et al. [36] phenotyped the infiltrated leukocytes from 20 primary resected NSCLC to assess the biological implications of the expression of three negative immune checkpoints PD-1, LAG-3 and TIM-3. A panel of 35 markers enabled the monitoring of 9 different immune populations: CD8^+^ T cells, CD4^+^ T cells, regulatory T cells (Tregs), natural killer (NK) cells, NKT cells, B cells, granulocytes, macrophages and dendritic cells. PD-1 was mostly expressed on CD8^+^ T cells, CD25^−^FOXP3^−^CD4^+^ T helper, CD25^+^FOXP3^+^CD4^+^ Tregs and CD3^+^CD56^+^ NKT cells, LAG-3 on CD8^+^ T cells and TIM-3 was broadly expressed with macrophages, NK and NKT cells showing the highest expression. Regarding their coexpression, 5.4% of the CD3^+^ TILs were positive for the three markers, 9.1% were PD-1^+^LAG-3^+^, 21% were PD-1^+^TIM-3^+^ and 10.3% were LAG-3^+^TIM-3^+^. Interestingly, it was demonstrated that PD-1 and TIM-3 coexpression favoured the persistence of exhausted T-cells [37]. As the panel contained markers indicative of cell functions, they investigated the functional characteristics of the T cells expressing the above-mentioned immune checkpoints and observed that their coexpression was associated with higher expression of functional markers. PD-1^+^LAG-3^+^TIM-3^+^ T cells showed the highest expression of activation markers CD69 and 4-1BB, cytotoxicity marker granzyme GZMB, proliferation marker Ki67, apoptotic receptor FAS and pro-apoptotic protein BIM. Finally, they evaluated the impact of the three immune checkpoints on the survival of 90 patients treated with PD-1 inhibitors. Neither high PD-1 nor high TIM-3 T cell expression was predictive of an improved PFS; however, patients expressing high LAG-3 showed a significantly poorer outcome and patients with low expression of LAG-3 and high expression of PD-L1 had an improved PFS. These results suggest that LAG-3 could be involved in resistance to immunotherapy and could be targeted to improve ICI response. Accordingly, combined therapy of anti-LAG-3 relatlimab and anti-PD-1 nivolumab had greater results on PFS in treatment-naïve patients with advanced melanoma compared with nivolumab monotherapy [38].

The large panels used in mass cytometry give the possibility to map the relationships between different cell populations and shift the focus on other cell types that enhance anti-tumor response such as CD4^+^ T cells. Tay et al. [39] reviewed the multiple lines of evidence suggesting their implication in anti-tumor immunity. Blood samples from NSCLC patients treated with nivolumab revealed that Tregs were more present in the blood of non-responders [40], consistent with Kamada et al.’s [41] study that demonstrated the dampening effect of Tregs on anti-PD-1 response. Besides, responders had higher frequencies of CD62L^low^ CD8^+^ and CD4^+^ T cells [40], the latter containing effector and effector memory T cells crucial for the establishment of an effective and sustained anti-tumor response [42]. The authors performed mass cytometry to better characterize this population and identified CD62L^low^ CD4^+^ T cells as T-bet^+^CD27^−^FOXP3^−^CXCR3^+^ Th1 cells that correlated with cytotoxic CD8^+^ T cells and PD-1 expression on CD8^+^ T cells. CD62L^low^CD8^+^ T cell subset was significantly increased in responders, although not as robustly as the CD62L^low^CD4^+^ T cell population. The monitoring of the CD62L^low^CD4^+^ T cell population before and after nivolumab treatment showed that long-term responders without disease progression in the 500 days after immunotherapy had higher frequencies of CD62L^low^CD4^+^ T cells before treatment compared to short-term or non-responders. In addition, patients with an ongoing response between 12 to 92 weeks post-nivolumab had significantly higher frequencies of CD62L^low^CD4^+^ T cells compared to patients who acquired treatment resistance. Notably, Liu et al. [43] also found evidence of Th1 expansion in responders after immunochemotherapy. The authors translated their findings into a predictive score, which could be used in a clinical setting. This predictive score was based on the percentages of CD62L^low^ and CD25^+^FOXP3^+^ among CD4^+^ T cells and significantly discriminated responders from non-responders after nivolumab treatment with a sensitivity of 92.9% and a specificity of 72.1% in a validation cohort of 86 patients. Hence, this study demonstrated the implication of CD4^+^ T cells in the maintenance of a sustained response after nivolumab. Immunomonitoring the CD62L^low^ CD4^+^ T cell subset in the clinic could be a useful tool to predict long-term responses.

It can be objected that mass cytometry is still expensive and time-consuming for routine use. Nevertheless, this technique can be used in exploratory studies before clinically validated methods in transitional research settings, as exemplified recently. Newel’s team demonstrated the heterogeneity of TILs, containing bystanders and tumor-specific CD8^+^ TILs characterized by high CD39 expression using mass cytometry. Notably, CD39^+^CD8^+^ T cells were predictive of response to immunotherapy in NSCLC patients [44, 45]. Therefore, they compared this technique to other clinically relevant methods for an accurate quantification of this population. Frequencies found with multiplex immunohistochemistry (mIHC) significantly correlated with mass cytometry data. Among patients treated with PD-1/PD-L1 inhibitors, responders had higher proportions of CD39^+^CD8^+^ T cells compared with non-responders whereas neither CD39^+^ cells nor CD8^+^ T cells retained significance.

### *scRNA-seq*

scRNA-seq made possible the investigation of tumor heterogeneity in an unbiased manner as it does not require the ‘markers selection’ step of mass cytometry. With the identification of patterns of expression programs, not only do we access cell-type information but also cell-state across patients [46].

Cytotoxic CD8^+^ T cells are thought to be the main drivers of anti-tumor immunity and the cornerstone of cancer immunotherapy response [47]. Gueguen et al. [48] questioned the origins of CD8^+^ TILs in NSCLC and conducted a pseudotime analysis. Trajectory inference algorithms use single-cell data to predict cell fate over time, giving insights into a dynamic process that can hardly be witnessed [49]. They identified blood circulating memory-like precursor states GZMK^+^ and KLF2^+^ CD8^+^ T cells recruited at the tumor site and tissue-resident precursors XCL1^+^CD8^+^ T cells. Results suggested that these precursors converted into a transitional state GZMH^+^CD8^+^ T cells to become terminally differentiated CD8^+^ T cells with a dysfunctional/exhausted phenotype. The late CD8^+^ T cells were found to be the most cycling cells compared with precursors. PD-1^+^TIM-3^+^CD39^+^CD8^+^ T cells were Ki67^+^ and comprised a CD103^+^ fraction that expressed PD-L1 whereas PD-1^−^TIM-3^−^ or PD-1^+^CD8^+^ T cells were Ki67^low^. These results were consistent with the TCR sequencing analysis where precursors shared many TCRs with GZMH^+^ and terminally differentiated CD8^+^ T cells. Besides, cycling cells TCRs preferentially overlapped with late CD8^+^ T cells, suggesting that the latter expand in response to tumor-antigen stimulation. Interestingly, they applied response signatures from Sade-Feldman et al. [50] that focused on melanoma patients treated with immunotherapy. KLF2^+^ and XCL1^+^ CD8^+^ T cells had an increased score for the good response signature; meanwhile, GZMH^+^ and terminally differentiated CD8^+^ T cells had an increased score for the poor response signature, consistent with the study of Guo et al. [51]. A similar model has since been proposed by Liu et al. [43] based on patients treated with immunochemotherapy. They developed the ‘clonal revival’ theory where pre-existing precursors locally expand and additional peripheral T cells are recruited at the tumor site to actively participate in the anti-tumor response following treatment as hypothesized in Wu et al.’s [52] paper, providing an explanatory mechanism of response to immunotherapy. Their results suggested that the tumor microenvironment promoted CXCL13 expression on CD8^+^ T cells whereas Banchereau et al. [53] described tissue-resident memory T cells (T_RM_) expressing high levels of CXCL13 transcript. T_RM_ are characterized by CD103 surface protein expression and their accumulation is promoted by TGF-β1 in mice [54]. Their presence in tumor infiltrate is associated with improved prognosis [54]. Inflamed NSCLC were shown to overexpress CD103-encoding gene ITGAE compared with desert or immune-excluded tumors and its expression on CD8^+^ T cells was positively correlated with PD-L1 expression [53]. T_RM_ expressed CXCL13, the immune checkpoints LAG3, PDCD1 (PD-1), HAVCR2 (TIM-3), TIGIT, CTLA4, a regulator of tissue-residency ZF683 and markers of tumor-specific response TOX [55] and ENTPD1 (CD39), consistent with Simoni et al.’s [44] study. These results strongly suggest that previously labelled exhausted CD8^+^ T cells were T_RM_. Additionally, single-cell TCR clonality analysis revealed that T_RM_ shared most clonotypes with MKI67^+^ cluster, suggesting that it could result from a proliferation of T_RM_. Based on OAK trial data [56], they could demonstrate the predictive value of high ITGAE expression with improved OS for patients treated with atezolizumab compared with patients receiving docetaxel. Interestingly, CXCL13 is also involved in the formation of tertiary lymphoid structures (TLS). TLS arise around inflamed tissues and resemble secondary lymphoid organs [57]. They participate in anti-tumor immunity by increasing antigen presentation and cytokine-mediated signalling and improve ICI response in melanoma [58], which was also recently demonstrated in NSCLC [59]. Besides, Patil et al. [54] identified a plasma cell signature that could significantly predict increased OS in patients receiving immunotherapy. mIHC revealed that plasma cells were located at the vicinity of TLS, which presence was also associated to improved survival. Importantly, compared to known biomarkers such as PD-L1 or tissue TMB, the plasma cell signature still had the strongest impact on survival. It must be noted that MS4A1 (CD20) expression correlated with PDCD1 (PD-1) and CD274 (PD-L1) in the study of Chen et al. [60]. However, B cell signatures did not statistically improve the OS of atezolizumab-treated patients in the study of Patil et al. [54].

One major pitfall of scRNA-seq data analysis is the annotation of cell populations. Multiple datasets are often combined and transcriptomic findings are complemented by mass cytometry to validate protein expression levels on specific cell populations. However, it requires additional work and technical issues of combining two different approaches make it more complex. To circumvent this limit, multimodal single-cell technologies have been developed. Notably, CITE-seq allows simultaneous single-cell profiling of both transcripts and surface proteins by using sequenceable DNA oligonucleotides conjugated to antibodies that can bind to epitopes displayed on the cell surface [61]. Leader et al. [62] identified a ‘lung cancer immune activation module’ (LCAM) predictive of immunotherapy response. Based on the POPLAR trial results [25], they could determine that patients with increased frequencies of PD-1^+^CXCL13^+^ activated T cells, SPP1^+^ monocyte-derived macrophages, IgG^+^ plasma cells and a high TMB had an improved OS when treated with atezolizumab compared with docetaxel-treated patients. PD-1^+^CXCL13^+^ T cells could overlap with previously described subsets in studies by Banchereau et al. [53] and Gueguen et al. [48]. Notably, Chen et al. [60] found high expression of IgG encoding transcripts in plasma-like B cells which toxicity varied according to disease stage in culture with lung cancer A549 cell line. Activated T cells, including CD4^+^ and CD8^+^ T cells, were found to overexpress PD-1 and CD39 proteins and had an increased percentage of tumor-specific TCR clones. Interestingly, mIHC revealed that samples with high LCAM scores had plasma cells distributed around PD-1^+^ T cell aggregates or TLS made of PD-1^+^ T cells and CD20^+^ B cells, in line with other publications [59, 60]. It is possible to predict ligand-receptor interactions to better understand cell-to-cell communication within the tumor microenvironment. The study by Leader et al. [62] unveiled probable crosstalk between CXCL13^+^ T cells and CXCR5^+^ B cells, 41BBL^+^ B cells and 41BB^+^ T cells. Therefore, the presence of LCAM could translate an increased anti-tumor immune response that supports patients’ response to immunotherapy. CD274 (PD-L1) gene expression poorly correlated with LCAM score contrarily to TMB, underscoring the necessity to select better predictive tools that include both tumor intrinsic and immune cell infiltrate features.

Lau et al. [63] demonstrated that NSCLC tumor cells expressed human leucocyte antigen (HLA)-II transcripts, mainly HLA-DRB1. Besides, they identified a cluster of cytotoxic GZMB CD4^+^ T cells that expressed high levels of PDCD1 and CTLA4, suggesting that this population could be involved in the anti-tumor response after immunotherapy. PD-L1 and HLA-II expression in classic Hodgkin’s lymphoma (cHL) were predictive of an improved outcome after immunotherapy [64]. As cHL expresses little HLA-I molecules, it emphasizes the possibility of an anti-tumor immunity independent from HLA-I antigen presentation. In Lau et al.’s [63] study, mIHC analysis indicated that cytotoxic CD4^+^ T cells potentially interact with HLA-II-expressing tumor cells. The cytotoxic T cells had the greatest clonal expansion, again indicative of a tumor-specific immune response. Notably, high PD-L1 expression on the tumor was not associated with a longer time to progression. Based on a retrospective cohort of 123 individuals, the authors developed a predictive model, based on TMB and a cytotoxic score calculated by taking the mean of log-transformed expression of 25 cytotoxic-related gene signature (NKG7, CXCL13, GZMH, HAVCR2, CCL5, GZMK, CCL4, GZMA, CCL3, CST7, CCL4L2, ACP5, TNFRSF9, TIGIT, GZMB, PDCD1, PRF1, LYST, SIRPG, LAG3, CARD16, TUBA4A, PTMS, CD74, KLRD1) highly expressed in both CD4^+^ and CD8^+^ cytotoxic T cells, that could significantly differentiate patients with long and short time to progression after immunotherapy, regardless of HLA-I deficiency. Curiously, they also identified two cytotoxic CD8^+^ T cell clusters CD103^+^CD39^+^ that expressed negative immune checkpoint transcripts PDCD1, LAG3 and TIGIT that resemble and could potentially overlap with the CD103^+^ and the CD39^+^ CD8^+^ T cells previously mentioned [44, 45, 53]. Besides investigating immune populations involved in response to immunotherapy, scRNA-seq can also help to characterize immune cells that trigger treatment resistance. Serine threonine kinase LKB1 mutated tumors are known to poorly respond to immunotherapy [65]. Transcriptional profiling of STK11/LKB1 mutant mouse models revealed the presence of hypofunctional CD8^+^ T cells with a decrease in memory-like CD8^+^ T cells and an enrichment in M2-like macrophages [66, 67]. Furthermore, the level of lactate was increased in STK11/LKB1 mutant patients, which could explain the immunosuppressive environment [68]. The blockade of the lactate pathway through MCT4 knock-out or the inhibition of Axl in DCs sensitized LKB1 mutant tumors to immunotherapy, suggesting that combination therapies could circumvent treatment resistance in LKB1 mutant NSCLC patients.

### *High-dimensional imaging and spatial transcriptomics*

Even though cell-to-cell interactions can be computationally predicted from scRNA-seq data, they are based on pairwise ligand-receptor expression; we therefore have no evidence of their actual behaviour in vivo. Imaging mass cytometry (IMC) can solve this problem and provide precious information on the NSCLC tumor architecture and immune cell crosstalk [69, 70]. Jia et al. [71] reported the first case of pseudoprogression after neoadjuvant immunochemotherapy. IMC enabled them to witness a shift from immune desert to infiltrated tumor that could have caused the enlarged lesions. The results showed cell-to-cell contact between CD8^+^ T cells and CD14^+^ and CD16^+^ monocytes, consistent with Leader et al. [62] predictions that involved IL-10, CCL3 and TNF receptors.

Another limit of scRNA-seq is the loss of spatial information regarding gene expression. Spatial transcriptomics enable us to analyse transcriptomic regulation in a tissue context while preserving the overall architecture of the sample [72]. The role of tumor-associated macrophages (TAMs) in promoting tumor growth and resistance to treatment is well known [73]. Larroquette et al. [74] demonstrated the predictive value of TAMs infiltration for NSCLC patients treated with immunotherapy. Indeed, patients with low CD163^+^ cell density within the tumor had improved clinical outcomes after immunotherapy. However, CD163^+^ cell infiltration in the stroma was not associated with the outcome, underscoring the importance of spatial information. The highly infiltrated tumors upregulated ITGAM (CD11b), CD27 and CCL5, a chemokine involved in TAM recruitment, whereas tumors with low CD163^+^ infiltrate upregulated HLA-E and BLC2. Among patients with high infiltrate of TAMs, some showed good treatment response, which was associated with an upregulation of genes associated with M1 phenotype and IFN-γ signalling pathway.

Other emerging technologies are being developed as screening tools for biomarkers in NSCLC, notably digital spatial profiling. Fluorescence images from formalin-fixed paraffin-embedded tissue sections stained with oligonucleotide-conjugated antibodies simultaneously identify proteins or RNA expressed in user-defined compartments such as tumor cells, stroma and immune cells [75]. Several studies demonstrated the feasibility and potential of such approach in both research and clinical settings [76–78]. However, this promising technology is still in its early stage and needs to be optimized before being used on a larger scale.

## Contribution of single-cell technologies in the investigation of ICI-induced immune-related adverse events (irAEs) in NSCLC

The clinical benefits of ICI are limited by irAEs. Grade ≥ 3 irAEs occur in approximately 10% of NSCLC patients treated by ICI and warrant ICI suspension or permanent discontinuation [79, 80]. Chemotherapy combined with PD-1/PD-L1inhibitors is associated with a lower risk of severe irAEs than PD-1/PD-L1 inhibitors alone and the addition of CTLA-4 inhibitors increases the risk of grade ≥ 3 irAEs [81]. Although the development of irAEs correlates with improved efficacy of PD-1/PD-L1 inhibitors [82], ICI-related pneumonitis and myocarditis of any grade have been associated with decreased survival in NSCLC patients [83, 84]. IrAEs treatment mainly relies on corticosteroids, from low to high dosages according to severity. A study conducted on 2750 lung cancer patients treated by ICI showed that, of the 2% of patients with severe irAEs, 43% developed steroid-resistant irAEs [85]. Hence, the pathophysiology of these highly heterogenous toxicities requires further investigation to guide risk stratification of patients and management strategies and extend the clinical benefit of ICI [86]. Here, we will review the contribution of single-cell technologies to provide key insights into the mechanisms and potential biomarkers of ICI-related irAEs in NSCLC patients (Table 2).

**Table 2:** Summary of the reviewed studies using single-cell technologies for the investigation of ICI-induced irAEs in NSCLC

Reference	Authors	Single-cell technology	Samples	Number of markers	Number of cells	Type of cells	Key findings
[88]	Berner et al. (2022)	ScRNA-seq	*N* = 510 adenocarcinoma	-	nd	CD3^+^CD8^+^CD137^+^ T cells	Napsin A–specific CD8^+^ IFN-γ^+^ T cells are more frequent in ICI responders and might drive irAEs
[89]	Wang et al. (2021)	Mass cytometry	*N* = 6 synovial fluid samples from ICI-related arthritis	n.d	42,144	CD8^+^ T cells	Clonally expanded CD38^hi^CD127^−^CD8^+^ T cells drive ICI-related arthritis
		scRNA-seq	N = 4 synovial fluid samples and 1 tissue sample	-	18,472		
[92]	Kim et al. (2022)	scRNA-seq	*N* = 8 paired samples of synovial fluid and peripheral blood	-	116,797	CD45^+^ cells	Circulating CXCR3^hi^CD8^+^ T cells are recruited into the joints via myeloid-secreted cytokines and contribute to ICI-related arthritis Th17 signature is enriched in synovial fluid from ICI-related arthritis after combined immunotherapy
		scTCR-seq					
[102]	Patel et al. (2022	Mass cytometry	*N* = 46 blood samples from NSCLCs patients	35–37	n.d	B cells	Decrease and functional impairment of Bregs in lung cancer patients increase the risk of developing severe irAEs after immunotherapy
[103]	Lou et al. (2022)	scRNA-seq	Blood samples from ICI-related myocarditis-suffering patients	-	17,729	CD45^+^ cells	Monocyte, NK cells and B cells promote inflammation during ICI-related myocarditis T cells express transcriptional programs related to anti-PD-1 resistance and hyperprogression Cell-to-cell communication is highly dysregulated

### *Mechanisms underlying ICI-related irAEs*

Although the pathophysiology of ICI-related irAEs resembles that of de novo autoimmune diseases, the precise underlying mechanisms have not been fully elucidated. Shared T cell clones were identified in matched ICI-related autoimmune skin lesions and tumor samples from NSCLC patients [87]. Besides, the discovery of tumor-associated self-antigens (Ag) combined with scRNA-seq further evidenced that autoreactive, napsin A–specific, CD8^+^ T cells are involved in both ICI efficacy and ICI-related inflammatory lung lesions [88]. This strongly suggests the existence of shared antigens and the capacity of ICI to simultaneously reinvigorate Ag-specific T cells in the two compartments. Besides reflecting treatment effectiveness, the identification of common pathways makes the occurrence of irAEs preventable.

Consistent with the previous notion, a study combining mass cytometry with scRNA-seq detected a predominant clonally expanded CD38^hi^CD127^−^CD8^+^ T cell population with effector and inflammatory phenotype driven by interferon in synovial fluid (SF) from ICI-related arthritis but not in SF from rheumatoid arthritis [89]. CD38^hi^CD127^−^CD8^+^ T cells from the peripheral blood of ICI-related arthritis were also increased, indicative of a systemic reaction to ICI rather than a local expansion [90]. Of note, CD38^hi^ CD8^+^ T cells reinvigorated by ICI have been detected, using mass cytometry, in tumor and peripheral blood samples from NSCLC patients, where they correlated with improved responses to PD-1 inhibitors [90, 91].

Strikingly, the same mechanism has been suggested in Kim et al.’s [92] publication with CX3CR1^hi^ and CXCR3^hi^ CD8^+^ T cells, two subsets that could largely overlap with CD38^hi^CD127^−^CD8^+^ T cells from the study of Wang et al. [89]. Although scRNA-seq data show little expression of CD38 transcript by CX3CR1^hi^ CD8^+^ T cells, the same was observed in CD38^hi^CD127^−^CD8^+^ T cells. Besides, CD38^hi^CD127^−^CD8^+^ T cells expressed high levels of CXR3 and CX3CR1. Kim et al. [92] took a step further with in silico cell-to-cell interaction analysis and in vitro migration assay suggesting that circulating CX3CR1^hi^ CD8^+^ T cells are recruited into the joints by CXCL9/10/11/16-secreting myeloid cells. These data indicate that Ag-specific T cells activated by ICI and recruited from the periphery may contribute to the pathogenesis of ICI-related arthritis in NSCLC patients.

scRNA-seq and scTCR-seq analysis were also conducted on colon samples from healthy individuals and melanoma patients with or without ICI-related colitis [93]. As in ICI-related arthritis, crosstalk between CXCR3^+^ T cells and CXCL9/10^+^ myeloid cells was proposed to be involved in the recruitment of T cells. However, as opposed to ICI-related arthritis, a large proportion of clonally expanded TCRs from T_RM_ were shared with cycling (MKI67^+^) effector (GZMB^+^ HLA-DRA^+^) CD8^+^ T cells in ICI-related colitis. The authors proposed that the differentiation of T_RM_ into effector CD8^+^ T cells, further evidenced by pseudotime analysis, could explain the early onset of ICI-related colitis. Tregs play an important role in preventing autoimmune disease and can be depleted by CTLA-4 inhibitors. Tregs expressing a Th1 profile (STAT1^+^ IL12RB2^+^ IL10^−^) were enriched in CTLA-4 inhibitors (± PD-1 inhibitors)-related colitis, compared with healthy colon tissue, whereas no Tregs depletion was observed [93]. Besides, Tregs from ICI-related arthritis were enriched and displayed enhanced immunosuppressive functions [92]. These data indicate that the increased risk to develop grade ≥ 3 irAEs following administration of CTLA-4 inhibitors, alone or in combination, is not caused by depletion of CTLA-4^+^ Tregs [94], but is rather caused by alterations in expression programs of suppressive CD4^+^ T cells [95]. Importantly, CD4^+^ Th17 cells, but not Th1 cells, persisted in the peripheral blood of patients with ICI-related arthritis following steroid administration, suggesting a role of the former cell population in irAEs resistance to steroids [92].

### *Potential biomarker of ICI-related irAEs*

Known risk factors of ICI-related irAEs in NSCLC patients include HLA-I homozygosity [96], BP180-specific immunoglobulin G [97], baseline levels of peripheral CD8^+^ T cells [98] and gut microbiome [99, 100]. The authors correlated the previously mentioned risk factors with improved responses to ICI. However, it is necessary to dissociate irAEs biomarkers from the efficacy of ICI, as the two events are closely related [101]. Mass cytometry analysis of peripheral blood samples from 46 NSCLC patients, prior to PD-1 inhibitor administration, identified 7 phenotypically distinct populations of regulatory B cells (Bregs) [102]. B-cell cytokine production was impaired in ICI-related irAEs patients, notably interleukine-10 (IL-10), a cytokine produced by Bregs with immunosuppressive properties. Consistent with this finding, the Bregs clusters from ICI-related irAEs patients were decreased and their defect participated in the emergence of ICI-related irAEs. Conversely, scRNA-seq analysis of peripheral blood samples from lung cancer patients revealed increased B cells, with substantially different B cell receptors repertoire, during ICI-related myocarditis remission vs onset [103]. Although changes in peripheral B cell populations were also associated with the development of severe ICI-related irAEs in melanoma patients, the incriminating phenotypes were different from those described in lung cancer patients [104]. This inter-tumor variability adds a layer of complexity to the study of ICI-related irAEs. Indeed, several studies have demonstrated that single-cell technologies are suitable tools to highlight rare events such as peripheral type-1 conventional dendritic cell [105], activated effector memory CD4^+^ T cell [106] and multimodal molecular states [107] as potential biomarkers of irAEs, dissociated from the response to ICI, in hepatocellular carcinoma, melanoma and metastatic thymic cancer patients, respectively.

## Conclusion

Single-cell technologies have led to an unprecedented depth of analysis of the tumor microenvironment, paving the way toward an exhaustive comprehension of the mechanisms of action and resistance to ICI. Indeed, these multiparametric approaches provide highly resolutive data to monitor phenotypic and functional heterogeneity, and refine our knowledge on immune cell composition. New cell types supporting or dampening ICI response are being identified and changes occurring during ICI treatment help patients’ stratification.

This level of information cannot be captured by low-resolution techniques, which barely allow for the rough identification of immune populations and usually include a very limited number of functional markers. This major limitation applies to most techniques that are validated for clinical applications. Therefore, if high-throughput technologies are currently used for screening candidate biomarkers among thousands of immune subsets, we should see the premises of their use for clinical applications within the next coming years. Indeed, the high cost of these technologies, which currently prevents their use in clinical routine, is expected to decrease with barcoding and cell hashing. In addition, this cost must be weighed against the clinical benefit of a therapy better adapted to a patient’s immune profile, and against the cost of monoclonal antibody therapies whose efficacy is bound to be low or non-existent in certain subgroups of patients [9, 10]. This question is particularly relevant given the large number of ICI in phases II and III studies. Finally, the fact that the mechanisms of action and resistance are more complex than initially expected, as they involve a large number of immune subsets, makes it unlikely that a single parameter can accurately predict response to these drugs.

The major asset of immune biomarkers is their clinical actionnability, which opens perspectives of therapeutic intervention. Indeed, the identification of alternative immune checkpoints that are upregulated upon ligation of therapeutic monoclonal antibodies on their targets enables to highlight potential immunotherapy combinations. Upregulation of immune checkpoints such as LAG-3, TIM-3 and BTLA by immune cells has been described following PD-1, PD-L1 and CTLA4 inhibition and was linked with enhanced resistance to these drugs in NSCLC [108, 109]. Consistently, in the results of the phase II CITYSCAPE trial assessing the efficacy of tiragolumab in combination with atezolizumab, the simultaneous targeting of TIGIT and PD-L1 significantly enhanced survival in NSCLC patients, with a safety profile similar to that of atezolizumab alone [110]. Other applications are expected with the identification of immunosuppressive populations that interfere with T cell anti-tumor functions and involved in resistance to immunotherapy that may require depletion before treatment initiation.

The next hits are expected with spatially resolved multiplexed profiling approaches. Indeed, the spatial architecture of the immune microenvironment directly impacts cellular interactions, and has been linked with response to immunotherapy in renal cell carcinoma [111], melanoma [112, 113] and lung cancer [74]. Results of ongoing studies are eagerly awaited and should further enrich the overall picture of the determinants of responsiveness to ICI. The next challenges to overcome in the spatial analysis are yet to come, with needs for improvement of the RNA capture efficiency, as well as the resolution of spatial measurements, to better capture rare cell types while avoiding analysis of doublets.

To conclude, the accumulation of knowledge gained from these high-throughput technologies will help identify the next-generation predictive biomarkers of response to ICI and successfully meet the challenges in oncoimmunology.
